# Muscle fat infiltration in chronic kidney disease: a marker related to muscle quality, muscle strength and sarcopenia

**DOI:** 10.1007/s40620-022-01553-0

**Published:** 2023-01-31

**Authors:** Carla Maria Avesani, Aline Miroski de Abreu, Heitor S. Ribeiro, Torkel B. Brismar, Peter Stenvinkel, Alice Sabatino, Bengt Lindholm

**Affiliations:** 1 Division of Renal Medicine, Baxter Novum, Department of Clinical Science, Intervention and Technology, Karolinska Institute, M99, Karolinska Hospital University Hospital Huddinge, 14186 Stockholm, Sweden; 2 Post-Graduate Program in Nutrition, Department of Nutrition, Federal University of Santa Catarina, Florianopolis, Brazil; 3 Faculty of Health Sciences, University of Brasilia, Brasília, Brazil; 4 Research Center in Sports Sciences, Health Sciences and Human Development, CIDESD, University of Maia, Maia, Portugal; 5 Unit of Radiology, Department of Clinical Sciences, Intervention and Technology, Karolinska Institute, Stockholm, Sweden; 6 Department of Radiology, Karolinska University Hospital, Stockholm, Sweden; 7 Department of Nephrology, Parma University Hospital, Parma, Italy

**Keywords:** Chronic kidney disease, Dialysis, Sarcopenia, Myosteatosis, Muscle fat infiltration, Muscle quality

## Abstract

Muscle fat infiltration (MFI) also known as myosteatosis refers to any deposit of lipids found in the skeletal muscle. MFI is preferably assessed by image-based methods like computed tomography (CT), magnetic resonance image (MRI) and ultrasound, normally from muscle groups located in the legs, arms and in the trunk. MFI is understood as a marker of muscle quality, where a muscle with higher fat deposition has lower contraction power and capacity to produce force per unit of muscle mass. This concept supports the hypothesis that a decrease in muscle strength is not always explained by a decrease in muscle mass, but also by other factors, including lipid deposition in the muscle. In the general population, MFI is associated with older age, physical inactivity and with insulin resistance and inflammation. In chronic kidney disease (CKD), MFI has been associated with a decrease in muscle strength and impaired muscle quality as well as with metabolic abnormalities, cardiovascular disease and increased mortality. Interventions aimed at reducing MFI in CKD are incipient, but it seems that guided exercise can ameliorate muscle quality in patients on hemodialysis. The aim of this narrative review about MFI in CKD is to draw attention to a still not often addressed complication in CKD. We conclude that more studies are warranted to investigate mechanisms and factors promoting MFI in CKD. Thus, clinical trials aimed at understanding the type, frequency and intensity of exercise that can diminish MFI and improve the clinical condition of the patients are needed.

**Graphical Abstract:**

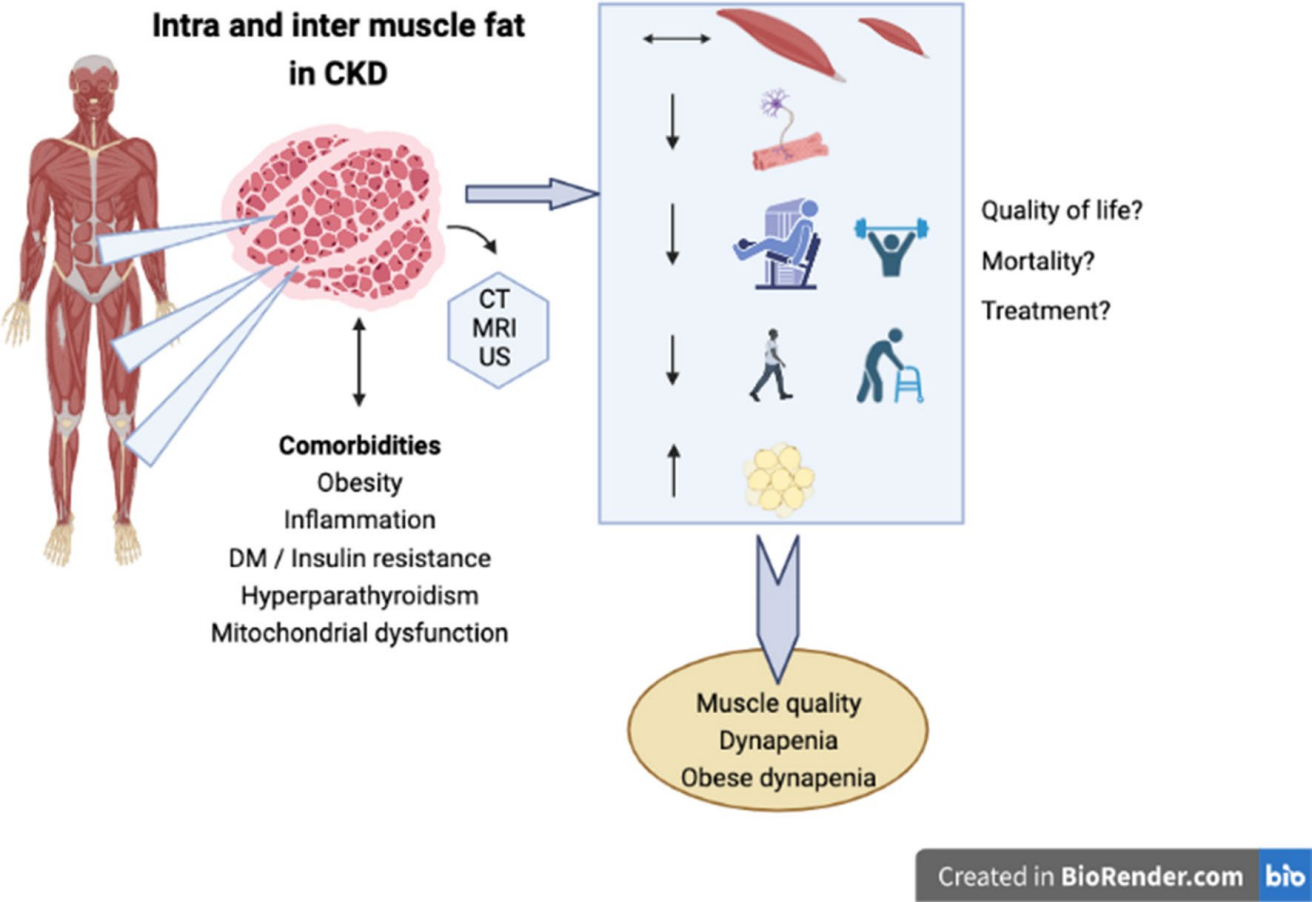

## Introduction

Patients with chronic kidney disease (CKD) undergo changes in body composition that have been documented in many studies since the 1960s [1–4]. These changes include an increase in body water content, a marked loss in muscle quantity (especially in patients on dialysis) and a decrease in body fat, although some patients on peritoneal dialysis might gain body fat during the first year of dialysis due to continuous glucose absorption from the dialysis fluid in the peritoneal cavity [5, 6].

The reason for this particular profile is likely explained by the metabolic alterations inherent to uremia, such as low-grade inflammation, insulin resistance, metabolic acidosis and secondary hyperparathyroidism that altogether pose a stimulus to protein degradation without a compensatory increase in protein synthesis [7]. Moreover, the sedentary lifestyle, the commonly observed insufficient energy and nutrient intake mainly in patients on dialysis, the dialysis procedure itself, and multiple metabolic and hormonal abnormalities further exacerbate the stimulus for protein degradation [8]. As a result, patients with CKD are generally exposed to a milieu characterized by a negative energy and protein balance, with a consequent gradual loss of skeletal muscle quantity and muscle strength, a condition known as sarcopenia [9]. Sarcopenia is a condition often observed in all CKD stages and it is associated with mortality in CKD stage 3 and 4 patients and in those on dialysis [10]. Concomitantly, losses in physical function (i.e., impairment of muscle strength and performance) have also been documented in CKD [11–13]. Studies performed in both non-dialysis and dialysis-dependent CKD have shown that handgrip strength, leg strength, and tests that evaluate physical performance (timed up-and-go, gait speed, short physical performance battery tests and others) are worse when compared to healthy individuals [13–15]. In addition, these markers of muscle function decrease over time and are associated with a worse quality of life, increased risk of falls, disability, frailty, hospitalization and mortality [13, 16, 17].

Notwithstanding, the decreased muscular strength and function in patients with CKD are disproportionate to the observed decrease in muscle volume [18, 19]. Moreover, studies have shown that patients with CKD also have a reduction in muscle quality, which translates into low muscle strength or physical performance per unit of muscle mass or volume [20]. This concept of low muscle quality is closely associated with muscle fat infiltration (MFI) [21, 22]. Fat infiltration changes the muscle structure composition and interferes with muscle contraction, thereby decreasing muscle strength and physical performance regardless of muscle quantity [21, 22]. Consequently, MFI and low muscle quality have emerged as two important concepts in the operational definition of sarcopenia in the revised European Working Group on Sarcopenia for Older People (EWGSOP2) statement [23]. In the EWGSOP2, low muscle strength is used to identify a state of probable sarcopenia, and, when combined with low muscle mass and/or low muscle quality, the diagnosis confirmed sarcopenia is made [23].

The current operational diagnosis of sarcopenia proposed by the EWGSOP2 marks a change in the concept of muscle abnormality that was previously limited to low muscle mass (or quantity) [24]. In fact, studies in older adults support the notion that there is a dissociation between the loss of skeletal muscle mass and strength [25, 26]. The missing piece of this puzzle may be MFI. In addition, MFI found within the muscle (intramuscular adipose tissue) is an ectopic fat, similar to visceral adipose tissue, that has been associated with metabolic changes such as inflammation, insulin resistance and dyslipidemia [21, 22, 27–29]. These findings have been reported mainly in studies on older individuals and in individuals with metabolic disorders, such as obesity and diabetes [28, 30–32]. MFI in CKD has been explored more recently but considering the plethora of muscle and metabolic abnormalities present in CKD, a better understanding of the clinical meaning of MFI should interest the nephrology community. This narrative review aims to explore the definition of MFI, the methods of assessment, the factors associated with this condition as well as its implications in CKD. Finally, recommendations of potential strategies to avoid and counteract MFI in patients with CKD are also discussed. The literature used for this review includes observational cohorts and cross-sectional studies, as well as experimental clinical trials.

## Muscle fat infiltration: definition and methods of assessment

Muscle fat infiltration (MFI) has been addressed in the literature using a variety of other terms, including intermuscular fat, intramuscular fat and myosteatosis [21, 22]. In general, MFI can be defined as any deposit of lipid found in the skeletal muscle tissue. However, there are three possible muscle sites where such deposits can be observed, and it is important to differentiate between them since they may exert different effects on muscle health. Fat deposits in muscle can be located as follows: (1) Beneath the muscle fascia and in-between muscle groups, known as intermuscular adipose tissue (IMAT); (2) In the extracellular adipose tissue within individual muscle fascicles, this location is named intramuscular adipose tissue, and; (3) As intracellular lipid vesicles, so-called intramyocellular lipids [22].

**Fig. 1:**
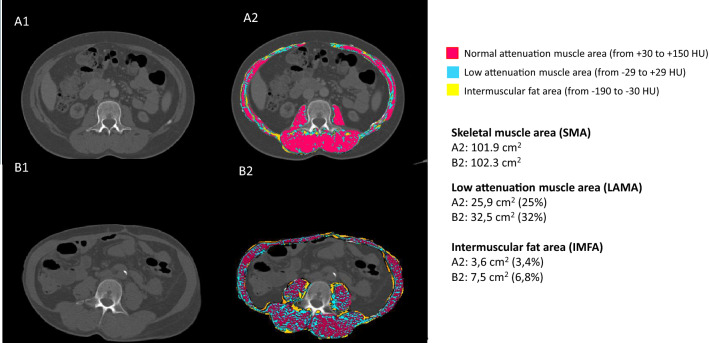
Representation of different levels of fat infiltration in skeletal muscle area assessed by computed tomography in the third lumbar vertebra in two female patients (age 43 years old) on hemodialysis. A1 and A2: images from the same patient before and after segmentation. B1 and B2: images from the same patient before and after segmentation. Patient represented in A2 has similar skeletal muscle area (SMA) to that of the patient in B2, but the patient in B2 has a higher proportion of low attenuation muscle area (LAMA) and higher intermuscular fat area (IMFA). The patient represented in A2 has similar SMA to that of the patient in B2, but the patient in B2 has a higher proportion of LAMA and higher IMFA, indicating lower muscle quality and higher muscle fat infiltration

In the research setting, MFI has been assessed in specific muscle groups, mainly in elderly and individuals susceptible to metabolic and muscle abnormalities, such as obesity, type-2 diabetes, and sarcopenia [21, 22]. The methods applied to measure MFI as well as their advantages and limitations are summarized in Table 1. The most often used methods are imaging techniques such as computed tomography (CT) and magnetic resonance imaging (MRI) which show good agreement when compared to muscle biopsy for the assessment of MFI [33, 34]. Muscle groups of major interest are in the lower limbs (quadriceps femoris, calf and tibialis anterior muscle) and at the level of the third lumbar vertebra (psoas, quadratus lumborum, abdominis, obliques and erector spinae muscle). Other methods can also be used, such as magnetic resonance spectroscopy, peripheral quantitative computed tomography and qualitative ultrasound [22]. Finally, as the gold standard, the muscle biopsy can be used for histological measurement of the adipose tissue located within muscle fibers. However, since muscle biopsy is a costly and invasive method, its use is limited to studies with small sample sizes that also aim to evaluate histological parameters such as muscle fiber type and mitochondrial dysfunction [35]. Among the above-mentioned methods, CT and MRI stand out for their good precision, high reproducibility, and ability to provide complementary information such as volumetry, in addition to MFI. The MFI found within the muscle (i.e., intramuscular adipose tissue) and beneath the muscle fascia (intermuscular adipose tissue) can be evaluated by CT, and the lipids within muscle cells (i.e., intramyocellular lipids) by MRI [33, 34]. The way MFI assessed by CT is reported varies from study to study. While some studies investigate intermuscular adipose tissue by applying the Hounsfield Unit (HU) range for the adipose tissue (− 190; − 30 HUs) within the total skeletal muscle area and then quantify the respective area, others identify IMAT by applying the HU range for muscle sites with low attenuation (− 29; + 29 HUs) named low attenuation muscle area (LAMA) and, normal attenuation (+ 30; + 150 HUs) called normal attenuation muscle area (NAMA) (Fig. 1) [22]. Since adipose tissue has low attenuation, muscle areas with more infiltrated fat are recognized as LAMA and signs for areas with high-fat infiltration and poor muscle quality. Muscle areas with less fat infiltration are recognized as NAMA, indicating low-fat infiltration and good muscle quality. Since IMAT may account for as little as 8% of the adipose tissue in the muscle, it probably represents a proportion of MFI that is too small to effectively evaluate, and likely underestimates differences in MFI between individuals [21]. For this reason, to avoid underestimation of MFI quantification, some studies report the muscle density of the total skeletal muscle area with LAMA and NAMA [36]. This approach allows evaluation of muscle quality (see example on Fig. 1). The variations in nomenclature and the variability in reporting the results of MFI makes comparisons across studies difficult. For clarity, in this review we will use the term MFI to generally describe myosteatosis. Otherwise, IMAT will be used for intermuscular adipose tissue and LAMA and NAMA when studies describe muscle attenuation.

**Table 1:** Methods applied for assessing myosteatosis

Method	Technology for the assessment of muscle fat infiltration	Advantages	Limitations
Computed tomography (CT)	Uses X-rays for an indirect measurement of MFI based on the tissue density—intra and intermuscular adipose tissue. The muscular tissue is divided into areas of normal or high-density lean tissue and areas of low-density lean tissue MFI can be reported by quantifying intermuscular adipose tissue area or volume, or by quantifying the area of muscle with low attenuation (LAMA), or the average skeletal muscle density to evaluate muscle quality Areas with high and normal attenuation muscle area (NAMA) indicate low MFI and therefore good muscle quality, while areas with low attenuation muscle area (LAMA) or low average muscle density are signs of high MFI and poor muscle quality In NAMA there is low fat infiltration but in LAMA, there is high fat infiltration (explaining the decreased tissue density) [22]	Noninvasive Good precision Images from the third lumbar vertebra can be used as an opportunistic assessment for already existing images from the trunk primarily acquired either for cardiovascular assessment or for tumor assessment It measures the total tissue volumes of skeletal muscle and the fat quantity in the assessed area	Radiation exposure The equipment has high operating costs and requires specialized personnel Analysis of muscle density may differ depending on the CT protocol and this affects the results Imaging with contrast media affects muscle density and may lead to errors when analyzing the image The procedure is time-consuming and it requires post-processing Availability of equipment is limited in most health centers
Magnetic resonance imaging (MRI)	Contrast images are determined by the chemical composition and microstructure of the tissue of interest, which directly affect frequency, density, diffusion, and relaxation properties of the detected nuclear spins. MRI uses the chemical properties of fat and muscle to directly measure the amount of IMAT within a body region [70]	No radiation exposure Noninvasive Preferable method for repeated measurements due to no radiation exposure Good precision It measures the total tissue volumes of skeletal muscle and the fat quantity in the assessed area	Very high cost The equipment has high operating costs and requires specialized personnel The procedure is time-consuming and it requires postprocessing The availability of the equipment is limited in most health centers
Magnetic resonance spectroscopy	It uses similar technology as MRI based on nuclear magnetic resonance. Identifies the proton resonances of the methylene (CH2) and methyl (CH3) groups of extramyocellular lipid stored in adipocytes and intramyocellular lipid stored in spherical droplets in the myoplasm [71]	Distinguishes between extra and intra myocellular lipids	High cost Time-intensive process
Peripheral quantitative computed tomography	To estimate the relative amount of muscle adiposity, muscle density is a hydroxyapatite-calibrated analogue of X-ray attenuation, which is validated to estimate muscle lipids and triglycerides. Muscle density combines all soft tissues between the inner edge of the subcutaneous fat and the outer edge of the bones as a composite index of intermuscular adipose tissue and myocellular adipose [34]	Noninvasive Lower radiation compared to CT Lower cost compared to the first three methods Portable equipment	Cannot clearly differentiate the individual muscle groups Limitations measuring some parts of the body, such as lower leg or arm
Ultrasound	Estimates muscle mass andtissue composition. Ultrasound can provide reliable measures of muscle thickness and echogenicity of a tissue in both the appendicular and axial skeletal muscles [72]. Echogenicity (ultrasound brightness) may be used to examine tissue composition [73]. Increased echogenicity indicates an increase in myosteatosis	No radiation exposure Lower cost compared to the first three methods Portable equipment	Intermachine and inter-operator variability Lack of a standardized approach for assessing myosteatosis
Muscle biopsy	Provides histological measurement of the adipose tissue located between the muscle fibers [21]	Gold standard method	Invasive

*CT* computed tomography, *MRI* Magnetic resonance imaging, *MFI* muscle fat infiltration, *NAMA* normal attenuation muscle area, *LAMA* low attenuation muscle area (NAMA)

One limitation for the assessment of MFI is the lack of normative tables to determine diagnostic cutoff points of myosteatosis, and another is the variability in the methods used for its evaluation. Recently, the CT scan of the trunk muscles at the level of the third lumbar vertebra of 20,664 healthy South Korean subjects aged 20–88 years old were used to determine sex-specific cutoffs for MFI diagnosis (31). They reported the area and average attenuation of the skeletal muscle for NAMA and LAMA separately. By using the cutoff points obtained with *T* score < − 2.0 for NAMA, myosteatosis was present in 5.9–8.8% in men and in 10.2–20.5% in women, depending on the age group. Moreover, the prevalence of myosteatosis increased with age, and was especially high in women ≥ 50 years old. In another study [37], tables with gender-specific percentiles were constructed for muscle radiation attenuation, skeletal muscle area, and skeletal muscle index (i.e., skeletal muscle area adjusted for height^2^) from the trunk muscles (also at the third lumbar vertebra) obtained from non-contrast enhanced CT scans. The study sample comprised 420 individuals from the Netherlands between 20 and 82 years old eligible for kidney donation. As in the study from South Korea, women had lower muscle radiation attenuation, indicating higher MFI. Moreover, they also observed decreasing muscle radiation attenuation with increasing age and BMI [37]. Population studies including other ethnicities are warranted to gather data on the percentile distribution of muscle radiation attenuation in different cohorts and for establishing appropriate cutoff values for the diagnosis of myosteatosis.

## Potential methodological errors in MFI assessment by CT and MRI

A great advantage of using CT to assess body composition in CKD is that CT performed for other reasons, such as unclear abdominal pain or evaluation prior to transplantation, can be analyzed retrospectively. However, clinical exams often include iodine contrast media to better visualize pathology. The use of contrast media causes an increase in fat and muscle attenuation which will result in an underestimation of MFI [38]. Because patients with CKD are more vulnerable to intravenous contrast media, the amount of contrast media often varies between the individual patients depending on their current kidney function and indication for the exam, making it difficult to make precise estimates of the induced error in a particular patient or scan. Modern CT protocols also often lower the tube potential to better enhance iodine and to reduce the contrast media dose. This will affect the attenuation of different tissues, resulting in an underestimation of muscle and fat area as well as of MFI [39]. The currently published data on MFI are most often based on a tube potential of 120 kV, but with modern CT scanning protocols they will more often be performed at 80 or 100 kV. Thus, to facilitate the comparison of different studies it is of great importance that studies specify the tube potential and contrast media protocol that has been used. Although not yet investigated, edema could be a cause of error for body composition assessment by CT. For MRI, scanner parameters such as field of view, pulse sequence and repetition time and echo time, scanner field strength and body temperature could theoretically influence the body composition analysis as well. Thus, investigations confirming these as the source of error for CT and MRI are warranted.

## Factors associated with MFI: from aging and sedentarism to clinical conditions

**Fig. 2:**
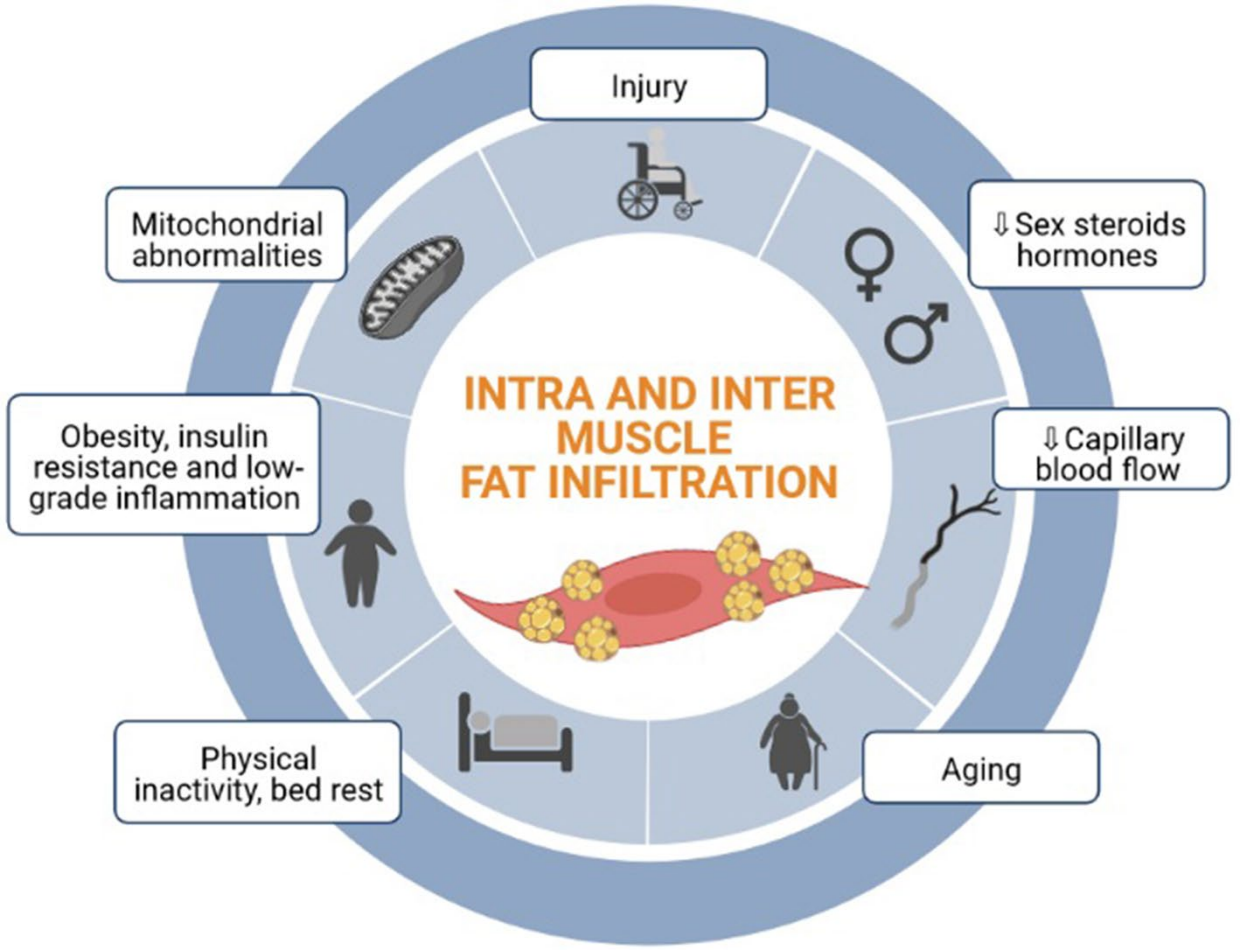
Factors associated with muscle fat infiltration

The causes of MFI are multifactorial and further studies should focus on the origin of increased fat infiltration. Presently, MFI is being described as a product of muscle injury, alterations of sex steroid hormones and capillary blood flow, aging, physical inactivity, obesity, and mitochondrial abnormalities (Fig. 2) [21, 22]. Some authors state that myosteatosis is an inevitable process with aging [25, 29, 40] and that in older individuals it occurs regardless of changes in body weight [25]. Delmonico et al. [25] followed individuals ≥ 70 years old for over 5 years, and reported that muscle volume and strength decreased over time while MFI increased, independently of changes in body weight. Another interesting finding was that over the 5-year period, the loss in muscle strength was two to five times higher than the loss in muscle volume. In addition, there was an age-related increase in MFI, and men with diabetes had a higher increase in MFI than those without diabetes, suggesting a metabolic association with MFI. Altogether, these findings show that in older individuals the loss of muscle strength is greater than the loss of muscle volume and that it coincides with an increase in MFI, suggesting a decrease in muscle quality [25]. Another study comparing young versus older adults also described that MFI is significantly higher in the elderly [41]. More recently, a large epidemiological study in Korean individuals (age range 20–88 years) that described MFI by CT, showed that the muscle areas with NAMA (low MFI and good muscle quality) decreased with age, while LAMA (high MFI and poor muscle quality) increased with age [36]. This age-related increase in MFI is likely due to the aging-related redistribution of adipose tissue, in which subcutaneous adipose tissue relocates and infiltrates the muscular tissue [21]. However, other studies have suggested that increases in MFI may be more closely related to muscle disuse, or inactivity, than aging per se [29, 42]. This implies that the cause of the increase in MFI in older adults is likely due to an association of factors related to the aging process and physical inactivity.

Other factors besides aging and physical inactivity that are related to MFI include obesity and metabolic derangements such as insulin resistance and low-grade inflammation. Maltais et al. [28] showed, in sedentary men, that LAMA (high MFI and poor muscle quality) of the abdominal and mid-thigh muscles was associated with higher fasting glucose, and impaired both the 2-h oral glucose tolerance test and homeostatic model assessment for insulin resistance (HOMA-IR) index. Therefore, it is not surprising that in individuals with type-2 diabetes, the proportion of LAMA was higher while that of NAMA was lower [30]. Moreover, in another report from the same study, the group made up of subjects having two or more components of the metabolic syndrome had a lower NAMA index (NAMA/total abdominal muscle), indicating lower muscle quality [31]. A recent review, including studies that quantified MFI, also showed that diabetics had higher MFI and that the MFI grade was associated with higher insulin resistance [27]. MFI has also been associated with low-grade inflammation [43]; this study comprising diverse groups of individuals showed consistent findings regarding the association between MFI and interleukin-6 (IL-6) [43].

One mechanism concerning the link of MFI with obesity and metabolic derangements may be that during weight gain, adipocytes may surpass their capacity to store fat, thereby increasing ectopic fat storage in non-adipose tissue such as in muscle, liver, and pancreas [22]. In the muscle, ectopic fat releases pro-inflammatory cytokines resulting in local tissue inflammation [21]. It is unclear to what extent MFI is merely a marker of metabolic dysfunction and to what extent it plays a significant role in insulin resistance and inflammation. One possibility, since MFI is close to the muscle fibers, is that the interaction between MFI, obesity, and metabolic derangements may amplify each other and lead to worsened muscle dysfunction and insulin resistance.

The factors associated with MFI and low muscle quality, such as obesity, insulin resistance, low-grade inflammation and dyslipidemia lead to cardiovascular disease (CVD). Not surprisingly, a longitudinal study that followed the participants for seven years showed that lower skeletal muscle density assessed in the calf by CT was associated with increased cardiovascular mortality in men aged 65 years and older [44]. Similarly, in another epidemiological study including adults (mean age 50.1 ± 3.6 years), higher abdominal MFI (reported as IMAT) was associated with higher coronary artery calcification [45]. Finally, in a cross-sectional study (mean age 55.6 ± 8.3 years), higher muscle quality (assessed by the NAMA/total abdominal muscle area ratio) was associated with better metabolic profile, including better blood pressure, glycemia, HOMA-IR, and visceral fat area, and less coronary artery calcification [46]. Although these are observational studies showing associations and not causality, collectively these findings suggest that good muscle quality (i.e., with low MFI) may be a protective factor for CVD.

In addition, MFI is a significant descriptor of physical function, indicating loss of strength and loss of muscle quality [47]. High levels of MFI (assessed as intermuscular adipose tissue) are associated with lower physical performance in studies with younger and older adults [41]. A study in which 18 healthy individuals underwent regional MRI of the thigh and calf for the assessment of skeletal muscle area and IMAT as a sign of MFI before and after a 4-week control period of usual activity and before and after a 4-week period of unilateral discontinuation of physical activity of the lower limbs, showed interesting findings [47]. No changes in muscle volume, subcutaneous fat and IMAT in the calf and thigh were observed after the 4-week control period, but after the 4-week limb activity discontinuation period, there was a decrease in calf and thigh muscle volume (by 7.9% and 7.4%, respectively), no significant changes in subcutaneous fat, and an increase in MFI of 20% in the calf and of 14.5% in the thigh. The loss of strength was greater in the thigh (of 20.4%) than in the calf muscles (reduction of 15%) and, in the regression model, the gain in MFI was related to the loss of strength. These findings support the hypothesis that physical inactivity is a cause of MFI and can partially explain the loss of strength and performance observed with aging. One theory behind it is that physical inactivity blocks the uptake of plasma triacylglycerol, alters lipid oxidation, and shifts the fuel metabolism from lipid to glucose utilization. This reduces intramuscular triacylglycerol mobilization and creates an environment that promotes MFI [48]. Therefore, a vicious cycle can be initiated where higher MFI can change muscle fiber orientation, decrease muscle elasticity, contraction, and power, and thereby decrease muscle strength. Of note, a study in older individuals (age 73.4 + 6.3 years) showed that after resistance training, only the participants with low MFI, as assessed by MRI quantification of IMAT, were able to improve muscle quality (meaning, better strength/unit of muscle mass), thus suggesting that MFI may impair improvement with resistance training in older adults [49].

**Fig. 3:**
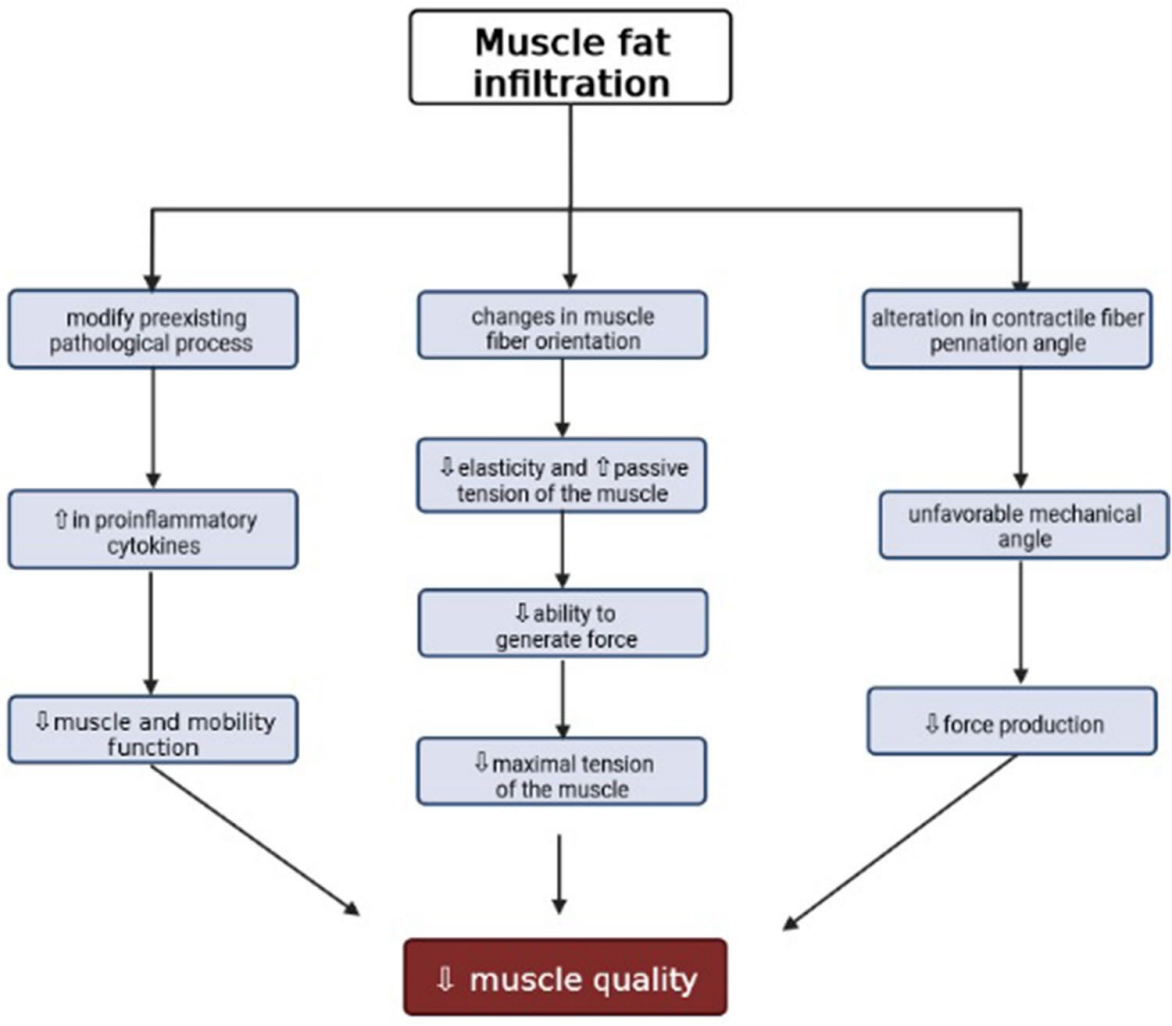
Mechanisms behind the increase in MFI and the decrease in muscle function with consequent decrease in measures of muscle quality

Although the association between high levels of MFI and decreased muscle function is consistent among studies, it is not known whether MFI is merely a marker of muscle dysfunction or whether it also has a direct effect on muscle dysfunction. Possible mechanisms and factors behind the decrease in muscle function with consequent decrease of muscle quality following an increase in MFI are illustrated in Fig. 3.

Finally, sarcopenic obesity, a condition in which a decrease in muscle mass and muscle strength occurs in combination with excessive body fat is also known to relate to MFI and poor muscle quality [50]. Individuals with sarcopenic obesity encompass a particular group that is prone to developing insulin resistance, sub-clinical inflammation, and chronic diseases [51]. In sarcopenic obesity, the deleterious metabolic effect of excessive body fat promulgates low muscle mass and strength, that altogether worsens muscle quality and sustains a cycle characterized by MFI, low muscle mass and strength, and metabolic derangements (mostly insulin resistance and low-grade inflammation) [51]. The combined condition of sarcopenia and myosteatosis was shown to be associated with increased mortality risk in patients with cirrhosis [52].

## Muscle fat infiltration in CKD: what do we know?

The study of MFI in CKD is still incipient and represents an important field to be explored. Table 2 summarizes the current findings in patients with CKD. The presence of MFI in patients with CKD was first described more than 30 years ago by Lindholm et al. [53], who compared muscle biopsies from the quadriceps femoris muscle in patients on peritoneal dialysis and healthy controls. Although this was a secondary finding in the study, it clearly showed that patients on peritoneal dialysis had twice as much MFI as healthy controls (116 ± 94 versus 66 ± 39 g of fat/kg muscle; *p* < 0.001) [53]. Later, Johansen et al. [20] described greater MFI as seen by MRI in patients on hemodialysis than in healthy controls of the same age and sex. In addition, it was noted that patients on hemodialysis had lower muscle contractile area and higher non-contractile area than healthy controls, but similar muscle cross-sectional area. Moreover, the ratio between maximal voluntary contraction per muscle area (kg/cm^2^) was lower in dialysis patients, suggesting lower capacity of the muscle to produce force and worse muscle quality [20]. Also in other diseases that feature increased protein catabolism and muscle abnormalities, such as in malignant tumors, MFI has been associated with lower muscle strength and mobility [54]. Similar to the findings observed in the general population, MFI, assessed by imaging techniques, or by the muscle strength/unit muscle area or mass ratio, has been shown to have profound negative effects on physical function [20, 55, 56], and has been associated with increased inflammatory markers [35, 55], insulin resistance [19], and mitochondrial dysfunction [35].

**Table 2:** Main characteristics and findings of studies assessing muscle fat infiltration in patients with chronic kidney disease

Author, year	Study design and patients	Methods	Aim	Main findings	Comments
Lindholm et al., 1986 [53]	Cross-sectional 33 CAPD patients (54 ± 13 years, range 29–76 years) 34 healthy controls (32 ± 2.1 years)	Muscle assessed: Quadriceps femoris Lipid content in the muscle Biopsy	To study the effect of CAPD on muscle water and electrolyte status in muscle biopsy	CAPD patients: 116 ± 94 g lipid/kg muscle Healthy controls: 66 ± 39 g lipid/kg muscle	Healthy controls were younger than patients and this may explain the finding of higher lipid content in the muscle of CAPD patients This was a minor finding in the study
Johansen et al., 2003 [20]	Cross-sectional 38 patients (55 ± 15 years) on dialysis 19 healthy controls (55 ± 13 years)	Muscle assessed: ankle dorsiflexor muscles Muscle cross-sectional area: MRI Muscle strength: Isometric dorsiflexor Muscle quality: Ratio muscle voluntary contraction /muscle cross-sectional area Physical performance: Gait speed	To quantify the extension of muscle atrophy in the lower limbs	Compared to healthy-controls, patients on dialysis had: ↓ muscle strength ↓ muscle contractile area ↓ gait speed, ↓ ratio muscle voluntary contraction /muscle cross-sectional area, Equal cross-sectional muscle area Lower gait speed was associated with lower muscle contractile area	This study did not report values of MFI, but showed that patients on HD had lower ↓ ratio muscle voluntary contraction /muscle cross-sectional area
Cheema et al., 2010 [55]	Cross-sectional 49 patients (62.6 ± 14.2 years) on HD	Muscle assessed: Thigh muscles Muscle cross sectional area: CT MFI: CT (average LAMA in HUs, and IMAT in cm^2^) Muscle strength: Isometric strength by Hip abduction, knee extension, triceps strength Physical performance: Gait speed, 6 min walk test	To investigate the association between etiological factors and lower muscle quantity and quality in patients on HD	MFI was directly associated with: Age, number of chronic diseases, and IL-8 MFI was indirectly associated with: Albumin, pre-albumin, physical functioning tests, muscle strength tests In a stepwise regression model, MFI was an independent determinant of gait speed and 6 min walk test	Muscle cross-sectional area, but not MFI was independently determinant of muscle isometric strength
Hui-Ling W et al., 2013 [19]	Cross-sectional 58 patients (53.8 ± 2.1 years) on HD 28 healthy controls (52.2 ± 4.9) years Sub-group of 6 patients on HD and 6 healthy controls underwent muscle biopsy	Muscle assessed: Thigh muscles Muscle cross-sectional area: MRI MFI: MRI (IMAT) Myocyte fiber morphology and lipid content: Biceps biopsy	To investigate the leg muscle quantity and quality including the muscle cross section area and MFI of patients undergoing HD	Compared to healthy controls, patients on HD: ↓ muscle cross-sectional area ↑ MFI ↑ ratio: MFI/muscle cross-sectional area contractile area Muscle biopsy: Patients on HD had atrophy of muscle fibers, and more lipid accumulation than healthy controls The ratio MFI/cross-sectional muscle area by MRI was positively associated with HOMA-IR index in patients on HD	
Wilkinson et al., 2018 [56]	Cross-sectional 61 patients with CKD not on dialysis (58.5 ± 14.9 years), eGFR: 31.1 ± 20.2 ml/min/1.73 m^2^)	Muscle assessed: Rector femoris Muscle cross-sectional area: Ultrasound MFI (increased echo intensity): Ultrasound Muscle strength: Lower limb strength by FysioMeter; Handgrip strength by dynamometer Physical performance: Gait speed, 6 min walk test, sit-to-stand 60 s test, incremental shuttle walk test, endurance shuttle walk test	To investigate the role of muscle quantity and quality on physical performance of non-dialyzed CKD patients	MFI was negatively associated with sit-to-stand 60 s and incremental shuttle walk test. No significant association was observed with lower limb strength and handgrip strength and gait speed In the regression models fully adjusted, muscle cross-sectional area was a significant predictor of sit-to-stand 60 s and incremental shuttle walk test; MFI was a significant predictor of sit-to-stand 60 s	MFI was a predictor of muscle strength and physical performance, but muscle cross-sectional area was the larger predictor of both markers of muscle function
Gamboa et al., 2020 [35]	Cross-sectional 20 patients with CKD not on dialysis (61 ± 9 years, eGFR: 14–60 mL/min/1.73 m^2^) 22 patients on HD (48 ± 12 years) 21 healthy controls (47 ± 10 years) Sub-group of 9 patients on HD and 15 healthy controls underwent muscle biopsy	Muscle assessed: Midthigh region Muscle cross-sectional area: MRI MFI: MRI Physical performance: 6 min walk test Mitochondrial function: Quadriceps muscle biopsy	To investigate the association between mitochondrial function and metabolic derangements in patients with CKD not on dialysis and on dialysis	Mitochondrial function was worse in patients with CKD not on dialysis and on HD as compared to healthy controls Mitochondrial dysfunction was associated with poor physical performance tests, MFI, inflammatory markers and oxidative stress	This study focused on studying factors associated with mitochondrial dysfunction
Keddar et al., 2020 [57]	Retrospective, longitudinal study. Median follow-up period for cardiovascular events and mortality events: 22 months 101 patients starting PD (56 ± 18 years) Reference values obtained from published data	Muscle assessed: Muscles from the area of the third lumbar vertebra Muscle area: CT, normalized by height MFI: assessed by CT and shown as muscle density (Hounsfield unit) Primary outcome: occurrence of non-fatal cardiovascular events (myocardial infarction or coronary revascularization, lower limb necrosis or peripheral arteryrevascularization)	To investigate the association between MFI marker and cardiovascular events in patients starting PD. They also investigated the determinants of muscle density in this patient group	58 patients had non-fatal cardiovascular events Patients with high muscle density (indicating lower MFI) had a lower number of cardiovascular events independently of traditional cardiovascular risk factors Multivariate regression analysis showed that older age, female gender, visceral fat area, low urine residual volume were determinants of lower muscle density attenuation (indicating MFI) In a subsample of patients that had kidney transplant and CT images (n = 12), muscle density increased (indicating a decrease in MFI) after restoration of kidney function	This study assessed MFI as muscle density and not as area or volume of MFI
Yajima T, 2022 [18]	Retrospective study. Patients with CT images with non-contrast. Follow-up of 7 years for mortality 217 patients (62.7 ± 13.7 years) on HD for 6 months	Muscle assessed: Muscles from the area of the third lumbar vertebra Muscle area: CT, normalized by height MFI: assessed by CT and shown as muscle density (Hounsfield unit)	To investigate whether skeletal muscle density (as a marker of quality) could accurately predict all-cause mortality in patients on HD	Skeletal muscle area and muscle density were positively associated with male sex, geriatric nutritional risk index, creatinine index and negatively associated with higher age and C-reactive protein In the adjusted analysis for confounding variables, lower muscle density but not lower skeletal muscle index was associated with higher all cause mortality risk	This study assessed MFI as muscle density and not as area or volume of MFI

*MFI* muscle fat infiltration, *CKD* Chronic kidney disease, HD hemodialysis, *PD* peritoneal dialysis, *CAPD* continous ambulatory peritoneal dialysis, *MRI* magnetic resonance image, *CT* computed tomography, *HOMA-IR* homeostatic model assessment for insulin resistance

In addition to the deleterious effects on physical function, as previously discussed, reduced muscle density is increasingly being linked to cardiovascular events and increased mortality risk [18, 57]. In a study comparing the effects of high MFI and low muscle mass on survival of patients on hemodialysis, skeletal muscle density, but not skeletal muscle index was an independent predictor of all-cause mortality [18]. This suggests that muscle quality, rather than muscle quantity, could have a more important role in predicting mortality. In another study, patients on peritoneal dialysis with low skeletal muscle density were shown to have increased risk for cardiovascular events [57]. In addition to factors well-known to be correlated to MFI, such as age, sex and abdominal adipose tissue, skeletal muscle density was also strongly correlated with reduced residual urinary volume, meaning that patients with worse renal function tended to have increased MFI [57]. Interestingly, in the subset of patients that went on to kidney transplantation, and consequently restoration of renal function and urinary volume, skeletal muscle density tended to increase, showing a reduction in MFI [57]. However, more studies are needed to better understand the causes and consequences of MFI in CKD.

Finally, it is important to note that the muscle area and the method of assessment varied among the studies. While some studies assessed MFI in the leg muscles [19, 20, 35, 58, 59], others assessed it in the muscles located in the area of the third lumbar vertebra [18, 57]. Whether the infiltration of fat in the muscle can be influenced by the area of assessment or rather if the metabolic effect of MFI differs depending on the area of assessment remains to be studied. In addition, the methods of assessment varied among the studies, ranging from muscle biopsy [53], to MRI [19, 20, 35, 57], to CT [18, 57, 58] or ultrasound [59]. The different methodologies applied made comparisons among the studies more difficult.

## Interventional studies to ameliorate the occurrence of muscle fat infiltration in CKD

Historically, exercise interventions for skeletal muscle mass have been primarily associated with high-performance sports and aesthetics [60]. However, in recent decades, a plethora of evidence has emerged about the crosstalk between skeletal muscle mass and other organs, such as the kidney and the adipose tissue [61, 62]. In the context of MFI, exercise has been documented as the main intervention to prevent and/or mitigate this condition [40]. Yet, as the assessment of MFI varies with the method of assessment, and since normative tables to diagnose myosteatosis are lacking, available evidence on the effects of exercise interventions to ameliorate MFI is heterogeneous. Different primary outcomes (e.g., IMAT, LAMA and NAMA) further add to the heterogeneity. In addition, there is little evidence on the role of exercise intervention on MFI in CKD.

Exercise is known as the most effective intervention for musculoskeletal health owing to the anabolic stimulus and adaptations following chronic training, especially due to the sensitivity to anabolic hormones (e.g., testosterone, insulin growth factor 1 (IGF-1), insulin, and growth hormone (GH)) [62]. As shown in Fig. 2, the main factors associated with MFI and low muscle quality are understood as factors that can be modified by exercise interventions. In a recent systematic review and meta-analysis, Ramírez-Vélez et al. [63] found a decrease in lipid infiltration and an increase in muscle radiation attenuation when comparing exercise interventions to a control group, but data were mainly related to women (84.7%) and may not be extrapolated to men. Moreover, Addison et al. [21] hypothesized that IMAT might be used as an energy source during skeletal muscle mass contraction promoted by exercise.

Although the structure and metabolic aspects of skeletal muscle quantity are of great importance for MFI, motor units and neuromuscular activation have been receiving attention due to their importance for producing force [64]. Thus, resistance/strength training seems to be the most appropriate exercise modality for attenuating MFI, since exercise induces greater and faster activation of motor units, especially those innervating type II fibers which are responsible for power and explosive contractions [65]. Despite exercise being recognized as the best intervention to prevent and treat physical impairments in CKD [66], which share common associated features with MFI, to date, there are only two studies that have also assessed the effects of exercise on muscle quality in patients with CKD [67, 68]. In the PEAK study conducted by Cheema et al. [67], patients on hemodialysis (*n* = 49, 62.6 ± 4.2 years) were randomized to usual care or to a progressive resistance training program consisting of two sets of 10 exercises at high intensity using free weights, three times per week for 12 weeks during the dialysis session. Results showed that there was a very small, but statistically significant increase of thigh muscle attenuation (indicating a decrease in IMAT) in the progressive resistance training group compared to the usual care (adjusted mean difference − 0.4 HU, 95% CI: -0.8 to 0.0 HU), with a moderate effect size (− 0.52, *p* = 0.04). The authors stated that such benefit could be explained by enhanced insulin sensitivity and through the synthesis of muscle fibers of higher integrity in which IMAT may be used as a substrate and oxidized. Contrary to these findings, Martin-Alemañy et al. [68], when randomizing patients on hemodialysis (*n* = 38, 34 ± 11 years) into two intervention groups (only oral nutritional supplementation, *n* = 14, and oral supplement with exercise, *n* = 10) for 6 months, showed a mild effect of the group receiving nutrition and exercise on ameliorating muscle quality assessed by muscle attenuation. Oral nutritional supplementation consisted of 434 kcal, 19.2 g protein, and 22.8 g lipids, while the exercise consisted of a progressive and personalized program that combined resistance/strength (4 exercises, 4 sets × 20 repetitions) and aerobic exercise (30 min, 12–13 rate of perceived exertion) during the hemodialysis session. After the 6-month intervention period, muscle attenuation revealed a borderline statistical difference favoring the nutrition with exercise group compared to the nutrition alone group (baseline: 52 ± 5.3 versus after intervention: 53 ± 3.7 and baseline: 54.6 ± 3.4 HUs versus after intervention: 56 ± 3.3 HUs, respectively, *p* value for intergroup differences = 0.054). The type of intervention did not exert an effect on changes in markers of physical performance. It can be hypothesized that the small sample size may be underpowered to detect changes in the outcome of muscle quality and physical performance. Of note, this study was made up of a group of young patients (34 ± 11 years) who mostly (87.5%) underwent hemodialysis twice a week [68]. Altogether, although the evidence in people with CKD is limited, considering the high prevalence of sedentary lifestyle in patients with CKD, especially in those on dialysis [69], and the catabolic milieu inherent to CKD [7], interventions designed to test how to ameliorate muscle health and reduce MFI are warranted.

## Summary and conclusions

The field of body composition assessment in CKD has been evolving over the last 4 decades. While the first studies focused on evaluating body fat and lean body mass to diagnose malnutrition or obesity, later studies focused on comparing reference methods with those that were more applicable to clinical practice for diagnosing nutritional status. Subsequently, the assessment of body composition has advanced and now includes evaluation of body fat distribution and ectopic fat in different tissues, including muscle. Regarding the latter, known as myosteatosis or MFI, much has been learned. First, MFI is a marker of muscle quality, with higher MFI indicating lower muscle quality. Second, lower muscle quality and higher MFI are associated with aging, lower physical performance and quality of life, inflammation and insulin resistance as indications of metabolic dysfunction. Finally, studies in CKD have already connected markers of MFI with markers of metabolic disorders, higher risk for CVD and mortality for all causes. This clearly shows that research on MFI can improve our understanding of the plethora of changes in the body composition in CKD patients and how these changes are related to lower quality of life and increased mortality risk. Lastly, interventions with programmed and supervised exercise aimed at ameliorating MFI have only just started and more clinical trials are needed to understand what type of frequency and intensity of exercise can diminish MFI and improve the overall clinical conditions (insulin resistance, inflammation, uremia) thus leading to better clinical outcomes (physical performance, quality of life and mortality). We hope that this first review of MFI in CKD may call attention to the need for studies of this important yet understudied complication in CKD.
